# Dietary patterns and chronic kidney disease risk: a systematic review and updated meta-analysis of observational studies

**DOI:** 10.1186/s12937-020-00661-6

**Published:** 2021-01-08

**Authors:** Ling-Qiong He, Xu-Hong Wu, Yi-Qian Huang, Xiao-Yan Zhang, Long Shu

**Affiliations:** 1 Department of Geriatric, The Third Affiliated Hospital of Zhejiang Chinese Medical University, No 219 Moganshan road, Hangzhou 31005 Zhejiang, People’s Republic of China; 2 Department of Endocrinology/Rheumatology and Nephrology, The Third Affiliated Hospital of Zhejiang Chinese Medical University, No 219 Moganshan road, Hangzhou 31005 Zhejiang, People’s Republic of China; 3 Department of Digestion, Zhejiang Hospital, No 12 Linyin Road, Xihu district, Hangzhou 310013 Zhejiang, People’s Republic of China; 4 Department of Nutrition, Zhejiang Hospital, Xihu district, Hangzhou 310013 Zhejiang, People’s Republic of China

**Keywords:** Dietary patterns, Chronic kidney disease, Systematic review, Meta-analysis

## Abstract

**Background:**

A number of studies have reported the association between dietary patterns and the risk of chronic kidney disease (CKD), however a consistent perspective hasn’t been established to date. Herein, we conducted this systematic review and meta-analysis of observational studies to assess the association between dietary patterns and CKD.

**Methods:**

MEDLINE, EBSCO and references from eligible studies were searched for relevant articles published up to 9 May 2020 that examined the association of common dietary patterns and CKD. The heterogeneity among studies was assessed by Cochran’s Q test and I^2^ methods.

**Results:**

Seventeen eligible studies, involving 149,958 participants, were included in our systematic review and meta-analysis. The highest compared with the lowest category of healthy dietary pattern was significantly associated with a lower risk of CKD (OR=0.69; CI: 0.57, 0.84; *P*=0.0001). A higher risk of CKD was shown for the highest compared with the lowest categories of Western-type dietary pattern (OR=1.86; CI: 1.21, 2.86; *P*=0.005). There were evidence of a lower risk of CKD in the highest compared with the lowest categories of light-moderate drinking pattern (OR=0.76; CI: 0.71, 0.81; *P*< 0.0001) and heavy drinking pattern (OR=0.67; CI: 0.56, 0.80; *P*< 0.0001).

**Conclusions:**

The results of this systematic review and meta-analysis show that a healthy dietary pattern and alcohol drinking were associated with lower risk of CKD, whereas a Western-type dietary pattern was associated with higher risk of CKD.

**Supplementary Information:**

The online version contains supplementary material available at 10.1186/s12937-020-00661-6.

## Introduction

Chronic kidney disease (CKD) affects between 11 and 13% of adults worldwide and is recognized as a major global health concern [1]. In the United States, CKD is a common noncommunicable chronic disease, with an estimated 26 million adults in 2007 [2]. In Brazil, the prevalence of CKD is approximately 9%, not including patients on dialysis [3]. In China, Zhang et al. reported that the overall prevalence of CKD surpassed 119.5 million (approximately 10.8% of the general population) in a nationally representative sample of Chinese adults [4]. Multiple risk factors for CKD, such as cardiovascular diseases, obesity, diabetes, smoking, and use of nephrotoxic medications have been well established [5]. In addition, dietary factors have also been acknowledged as the important risk factors for CKD [6].

In the past, some epidemiological studies particularly focused on dietary interventions as an important tool to prevent or slow down the adverse prognosis of CKD [7] and had well established the role of individual nutrients and foods and/or food groups on the risk of CKD [8, 9]. However, in reality, individuals do not eat nutrients or foods in isolation, but consume meals containing combinations of many foods and nutrients that possibly interact with each other [10]. Consequently, dietary pattern analysis is now widely accepted in the realm of nutritional epidemiology as a more recognizable approach for assessing the relationship between diet and diseases, because it takes into account the complexity of whole-diet and potentially facilitates nutritional recommendations [11].

Up to now, there have been considerable attentions in medical research on the relationship between dietary patterns and the risk of CKD [12–19]. However, these studies have yielded inconclusive results. Therefore, to clarify the potential associations between dietary patterns and risk of CKD, we performed an updated meta-analysis to synthesize the results of studies published up to 9 May 2020.

## Methods

### Literature search strategy

An electronic literature search was conducted throughout MEDLINE (US National Library of Medicine, Bethesda, MD) and EBSCO (Elton B. Stephens Company) for identifying human studies written in the English and Chinese languages published up to 9 May 2020, that included the following keywords or index terms: “nutrition” OR “diet” OR “dietary pattern” OR “eating pattern” OR “food pattern” OR “alcohol drinking” OR “alcohol consumption” AND “chronic kidney disease” OR “kidney disease” OR “End-Stage Renal Disease” OR “ESKD” OR “CKD”. In addition, we also manually searched the reference lists of the retrieved (including meta-analyses and brief reported) and gray literature for further eligible articles.

### Studies included criteria

Two independent authors (Shu L and He LQ) read the titles and abstracts of the articles retrieved in the initial search to identify studies that reported the relationship between diet and CKD. Any disagreements between two independent reviewers were discussed and resolved by consensus or by a third independent reviewer (Huang YQ) if necessary. All authors agreed on the relevant articles, then the full-text articles were reviewed against inclusion and exclusion criteria for the present systematic review and meta-analysis.

To be eligible, studies had to fulfill the following criteria: (1) The study was an observational study; (2) reported the association between dietary and/or food patterns and CKD risk; (3) Factor analysis, reduced-rank regression and/or principal component analysis were used to identify dietary and/or food patterns; (4) Odds ratios (OR), hazard ratios (HR) or relative risks (RR) and percentage of CKD (or sufficient information to calculate them) had been listed;(5) CKD diagnoses were confirmed by clinical interviews, or self-report on a previous physician-made diagnosis of CKD.

Dietary patterns are defined as the intake of foods that reflect habitual dietary intake. To minimize error, the two independent authors ensured that the selected food or dietary patterns were similar with regard to factor loadings of foods, which are consumed within those dietary patterns. For instance, the first pattern, named as a prudent/healthy pattern tended to have high loadings of foods, such as vegetables, fruits, whole grain, fish, olive oil, soy and antioxidants(e.g. Vitamin C and E, flavonoids, and carotenoids), and low fat dairy; The second pattern, named as a western-style pattern tended to have high factor loadings for foods, such as refined grains, red and/or processed meat, sweets, desserts, butter, fast food and soft drink; The third pattern, named as a drinker pattern tended to have high loadings of beers, wines, and white spirits.

### Definition of “high intake”

Dietary and/or food patterns were derived by using factor analysis and/or principal component analysis. Factor scores for each pattern were categorized into quintiles, quartiles, or tertiles (the lowest category and the highest category represent lowest and highest adherence to a specific dietary pattern, respectively.).The different forms of alcohol consumption were converted into grams of ethanol per day. Alcohol consumption of > 50 g/d (4 drinks) for men or > 25 g/d (2 drinks) for women was defined as a high consumption of alcohol or heavy drinking [20].

### Data extraction

Two independent reviewers extracted the following information from all eligible studies: first author, publication year, country, study design, sample size, number of CKD, dietary assessment method, naming dietary patterns and the variables that were adjusted for in our analysis.

### Assessment of heterogeneity

The Cochran’s Q statistic and *I*^*2*^ statistic were used to assess heterogeneity. A *P* value of Q-test > 0.10 or *I*^*2*^*<* 50% indicated an absence of heterogeneity between studies, and a fixed-effects model (Mantel-Haenszel method) was used to calculated the pooled ORs. If a *P* value of Q-test ≤0.10 or *I*^*2*^*≥*50% indicated a high degree of heterogeneity among studies, then a random-effects model (DerSimonnian and Laird method) was used [21].

### Quality assessment

The same two reviewers used the Newcastle-Ottawa Quality Assessment scale [22] to assess the methodological quality of the included studies in the present meta-analysis. This scale consists of three main domains (selection of participants, comparability of participants, and assessment of outcome/ exposure) and eight questions in total. These questions were assessed and each satisfactory answer received 1 point (may receive 2 points in comparability categories), resulting in a maximum score of nine. Only these studies which the majority of the questions were deemed satisfactory(e.g. with a score of 6 or higher) were considered to be of high methodological quality [23].

### Data analysis

The original studies reported the results of dietary pattern in terms of tertiles, quartiles and quintiles of dietary factor scores and the risk of CKD. We applied this systematic review and meta-analysis to assess the risk of CKD in the highest versus the lowest categories of healthy, Western-style and drinker dietary patterns. If original studies reported risk ratios (RR) or hazard ratios (HR) instead of OR, it was treated the same as OR when the reported prevalence of CKD was less than 20%. Multivariable adjusted OR, HR and RR with 95% CIs from individual studies were weighted and combined to produce an overall OR. Publication bias was assessed by inspection of the funnel plot and by formal testing for “funnel plot” asymmetry using Begg’s test and Egger’s test [24]. Sensitivity analysis was conducted to determine whether the differences in study design, sample size, age and race affected our study conclusions.

Statistical analyses were conducted with the use of the Review Manager, version 5.0 (Nordic Cochrane Centre Copenhagen, Denmark) and STATA, version 11.2 (STATA Corp, College Station, TX, USA). All *P-* values were two-tailed, and *P*-values< 0.05 were deemed as statistically significant.

## Results

### Overview of included studies for the systematic review and meta-analysis

**Fig. 1:**
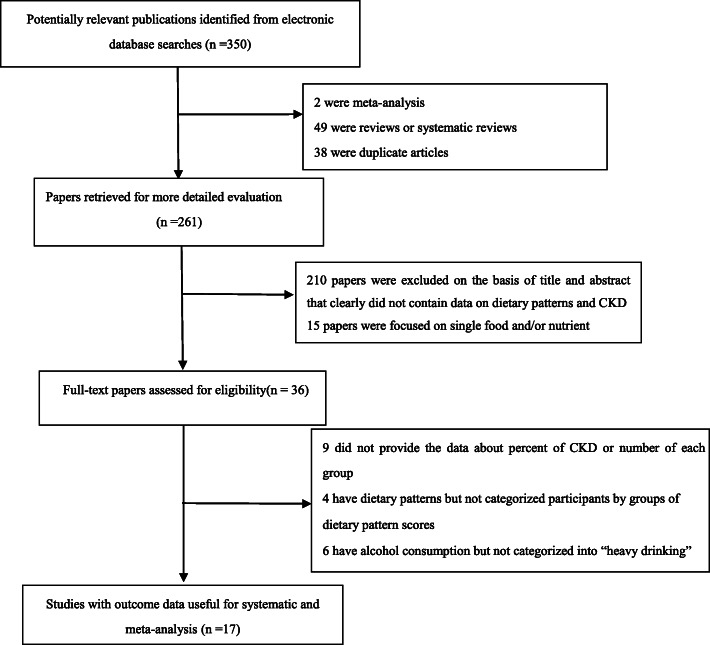
Flow chart of article screening and selection process

A literature search in the database of MEDLINE and EBSCO identified 350 studies, 333 of which were excluded based on the reasons listed in Fig. 1. Seventeen articles [12, 13, 19, 25–38] fulfilled the inclusion criteria and were included in this systematic review and meta-analysis, including 7 [12, 13, 19, 26, 27, 29, 32] cross-sectional studies, and 10 [25, 28, 30, 31, 33–38] cohort studies. Descriptive information for each included study in this systematic review and meta-analysis were shown in Table 1. Besides, the results from seventeen studies have been included in Table 2.

**Table 1:** Characteristics of 17 studies included in the meta-analysis (− 2020)

Author Publication Year	Location	Study design	Total number of subjects	Age	Diet-assessment method	Factors adjusted for in analysis	Dietary patterns identified
Mazidi et al. 2018 [12]	United states	Cross-sectional	21,649	≥18y	FFQ	Age, sex, race, body mass index, triglyceride, high density lipoprotein, diabetes,and hypertension	Saturated-MUFA; minerals and vitamins; Cholesterol-PUFA
Shi et al. 2018 [13]	China	Cross-sectional	8429	≥18y	FFQ	Age, gender, intake of energy,education, income, urbanization level, smoking, alcohol drinking, physical activity, overweight/obesity, hypertension, and diabetes.	Traditional southern; Modern.
Xu et al. 2020 [19]	China	Cross-sectional	2004	45-59y	FFQ	Age (continuous), gender, income (continuous), education level (<high school, high school, >high school), physical activity level (light, moderate, heavy), smoking status (never, current, former), BMI (continuous), T2DM(yes/no) and hypertension (yes/no), total energy intake (continuous)	Traditional southern Chinese; Western; Grains-vegetables
Yuzbashian et al. 2018 [25]	Iran	Cohort	5904	≥30y	FFQ	age (continuous), sex (male, female), smoking (never, current smoker), total energy intake (continuous), body mass index (BMI; continuous), angiotensin-converting enzyme inhibitor (ACEi; yes, no) and physical activity (low, moderate and high)	DASH-style diet
Kurniawan et al. 2019 [26]	China (Taiwan)	Cross-sectional	41,128	40-95y	FFQ	age, gender, BMI, smoking status, drinking status, physical activity, high cardiovascular risk profile, hypertension status (except hypertension variable), diabetes status (except diabetes variable), albumin, and CRP	Unhealthy
Eimery et al. 2020 [27]	Iran	Cross-sectional	266	60–83 y	FFQ	Age, sex, BMI, smoking status, physical activity, BP, use of medications, dietary intakes of protein, potassium, calcium, magnesium and phosphorous, supplement intake, socioeconomic status, and energy intake	Healthy; Western; Traditional
Asghari et al. 2018 [28]	Iran	Cohort	1630	≥27y	FFQ	Age, sex, smoking, total energy intake, physical activity, and body mass index, diabetes and hypertension	Lacto vegetarian; Traditional Iranian; Hiagh fat,high sugar
Paterson et al. 2018 [29]	United Kingdom	Cross-sectional	1033	56-100y	FFQ	Age, BMI, presence of diabetes, presence of hypertension, ever smoking, presence/history of ischemic heart disease, presence/history of cerebrovascular accident and ever alcohol.	Healthy;unhealthy
Lara et al. 2019 [30]	United states	Cohort	16,068	≥45y	FFQ	unadjusted	Plant-based; southern
Hu et al. 2019 [31]	United states	Cohort	12,155	45-64y	FFQ	Age, sex, race-center, total energy intake, education level, income, and estimated glomerular filtration rate, physical activity, smoking status, and pack-years, BMI, diabetes,systolic blood pressure, antihypertensive medication use, and HDL cholesterol. Dietary acid load	HEI-2015;AHEI-2010;aMed
Rouhani Hossein et al. 2019 [32]	Iran	Cross-sectional	221	Mean:56.5y	FFQ	Age, family history of colorectal cancer, history of endoscopy, physical activity, pack-years of smoking before age 30, race, aspirin use, total energy intake, and BMI	High fruits and vegetables; high simple carbohydrate and sugar; high fat
Huang et al. 2013 [33]	Sweden	Cohort	1110	Approximately 70 y	FFQ	BMI, physical activity, smoking status, education, hypertension, hyperlipidemia, and diabetes	Mediterranean Diet
Hu et al. 2020 [34]	United states	Cohort	12,692	45-64y	Questionnaire	Total energy intake, age, sex, race-center, income, education level, health insurance, smoking, physical activity, diabetes status, hypertension status, body mass index, baseline estimated glomerular filtration rate	drinker
Koning et al. 2015 [35]	Netherlands	Cohort	5476	28-75y	Questionnaire	Age, sex, height, weight, smoking status, parental history of CKD, history of cardiovascular disease, and educational level	Alcohol consumption
Sato et al. 2014 [36]	Japan	Cohort	9112	40-55y	Questionnaire	age, body mass index, systolic blood pressure, diastolic blood pressure,fasting plasma glucose, smoking habits (nonsmokers, past smokers, and current smokers), and regular leisure-time physical activity (yes/no).	Drinking pattern
Okada et al. 2019 [37]	Japan	Cohort	9116	40-55y	Questionnaire	Age, BMI, systolic blood pressure, diastolic blood pressure, fasting plasma glucose, smoking habits (nonsmokers, past smokers, and current smokers), and regular leisure-time physical activity (yes/no),eGFR at baseline	Drinkers
Foster et al. 2015 [38]	United kingdom	Cohort	1964	Mean:62.5y	FFQ	other listed lifestyle factors, age, sex, baseline eGFR, BMI, hypertension, diabetes, and dipstick proteinurial	Alcohol consumption

BMI: body mass index;DASH: dietary approaches to stop hypertension;BP: blood pressure; FFQ: food frequency questionnaire;T2DM:type 2 diabetes mellitus

**Table 2:** The results from 17 studies included in this systematic review and meta-analysis (− 2020)

Author/Publication Year	Countries/areas	Results
Mazidi et al. 2018 [12]	United states	Vitamins and trace elements intake are associated with lower risk of prevalent CKD.
Shi et al. 2018 [13]	China	Traditional southern dietary pattern is positively associated, and modern dietary pattern is inversely associated, with CKD among Chinese adults.
Xu et al. 2020 [19]	China	The Western pattern is associated with an increased risk, whereas the grains-vegetables pattern is associated with a reduced risk for CKD.
Yuzbashian et al. 2018 [25]	Iran	Higher adherence to the low-sodium DASH-style diet might be associated with a lower risk of incident CKD among high-risk adults.
Kurniawan et al. 2019 [26]	China (Taiwan)	The RRR-derived kidney function-related dietary pattern, characterized by high intake of processed and animal foods and low intake of plant foods, predicts the risks for developing cardiovascular disease and moderately/severely impaired kidney function among middle-aged and older adults.
Eimery et al. 2020 [27]	Iran	Higher adherence to the healthy dietary pattern may improve renal function while Iranian traditional pattern was associated with significantly increased odds of incident CKD and albuminuria.
Asghari et al. 2018 [28]	Iran	The high fat, high sugar dietary pattern was associated with significantly increased (46%) odds of incident CKD, whereas a lacto-vegetarian dietary pattern may be protective against the occurrence of CKD by 43%.
Paterson et al. 2018 [29]	United Kingdom	An unhealthy dietary pattern was associated with lower renal function and greater prevalence of chronic kidney disease.
Lara et al. 2019 [30]	United states	Adherence to a plant-based dietary pattern was inversely associated with eGFR< 60 ml/min/1.73m^2^, whereas the Southern dietary pattern was positively associated with eGFR< 60 ml/min/1.73m^2^.
Hu et al. 2019 [31]	United states	Higher adherence to healthy dietary patterns during middle age was associated with lower risk of CKD.
Rouhani Hossein et al. 2019 [32]	Iran	high fat dietary pattern was directly associated with progression of CKD.
Huang et al. 2013 [33]	Sweden	Adherence to a Mediterranean dietary pattern is associated with lower likelihood of CKD in elderly men.
Hu et al. 2020 [34]	United states	Consuming a low or moderate amount of alcohol may lower the risk of developing CKD
Koning et al. 2015 [35]	Netherlands	Alcohol consumption was inversely associated with the risk of developing CKD.
Sato et al. 2014 [36]	Japan	Among middle-aged Japanese men, the people who drank middle-range quantity, specifically who drank 4–7 days/week, had lower risk of CKD than nondrinkers.
Okada et al. 2019 [37]	Japan	Serum uric acid level and daily alcohol consumption were independently associated with the risk of CKD. Nondrinkers with the highest serum uric acid level had the highest risk of CKD
Foster et al. 2015 [38]	United kingdom	No associations were observed with physical activity, smoking status, or alcohol intake with incident eGFR < 60 or rapid eGFR decline (all *p* > 0.19).

### Healthy dietary pattern

**Fig. 2:**
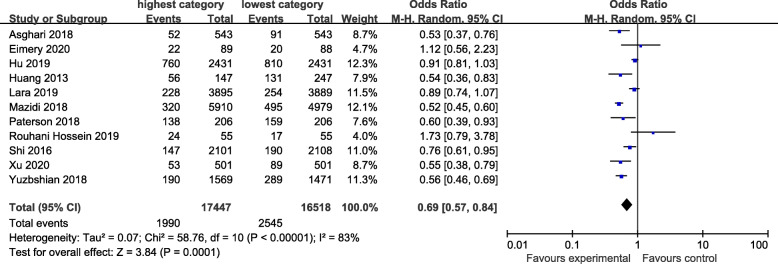
Forest plot for ORs of the highest compared with the lowest category of intake of the healthy dietary pattern and CKD

The healthy dietary pattern is characterized by high intake of vegetables, fruits, fish, low-fat milk and whole grains. The association between “healthy” dietary pattern and the risk of CKD was shown in Fig. 2. There was evidence of a lower risk of CKD in the highest compared with the lowest category of “healthy” dietary pattern (OR=0.69; CI: 0.57, 0.84; *P*=0.0001) where all studies were combined in the random-effects model. The heterogeneity was apparent in all the studies (*P*< 0.00001; I^2^=83%).

### Western-type dietary pattern

**Fig. 3:**
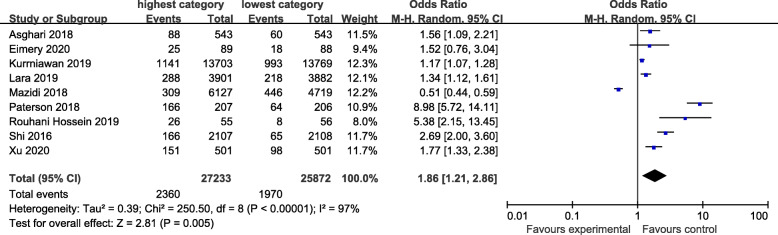
Forest plot for ORs of the highest compared with the lowest category of intake of the Western-type dietary pattern and CKD

The Western-type dietary pattern is characterized by high intakes of all kinds of red and/or processed meats, refined grains, sweets, high-fat dairy products and high-fat gravy. Figure 3 showed the association between western-type dietary pattern and the risk of CKD. There was evidence of a higher risk of CKD in the highest compared with the lowest category of western-type dietary pattern (OR=1.86; CI: 1.21, 2.86; *P*=0.005). A random-effects model was used to assess the data. The heterogeneity was apparent in all the studies (*P*< 0.00001; I^2^=97%).

### Drinking pattern

**Fig. 4:**
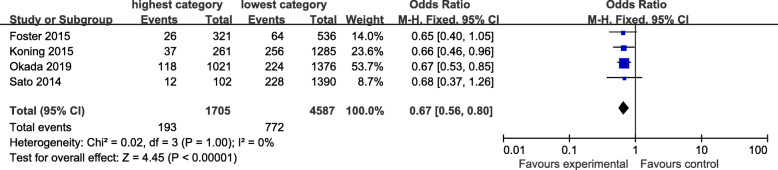
Forest plot for ORs of the highest compared with the lowest category of intake of the light-moderate drinking pattern and CKD

**Fig. 5:**
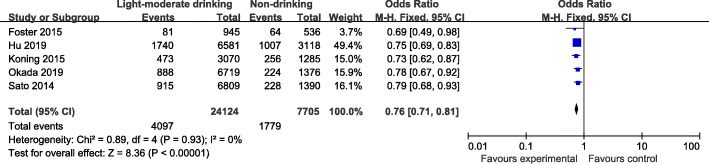
Forest plot for ORs of the highest compared with the lowest category of intake of the heavy-drinking pattern and CKD

The drinking pattern is characterized by high intakes of alcohol-containing beers, red wine, and white spirits. Four articles identified heavy drinking pattern in this meta-analysis. There was evidence of a lower risk of CKD in the highest compared with the lowest category of heavy drinking pattern (OR=0.67; 95%CI: 0.56–0.80; *P*< 0.0001) in Fig. 4. Data from these studies were assessed using a fixed-effects model, and there was no obvious evidence of heterogeneity(*P*=1.0; I^2^=0). Pooled results from five articles identified light-moderate drinking pattern. Figure 5 showed an obvious evidence of a lower risk of CKD in the light-moderate drinking compared with non-drinking (OR=0.76; 95%CI: 0.71–0.81; *P*< 0.0001). Data from these studies were assessed using fixed-effects model, and there was no obvious evidence of heterogeneity (*P*=0.93; I^2^=0).

### Publication bias

Visual inspection of the funnel plots revealed little evidence of asymmetry, and and thus little evidence of publication bias (highest compared with lowest category: healthy pattern *P*=0.205; western-type pattern *P*=0.314; heavy drinking pattern *P*=0.855; light-moderate drinking pattern *P*=0.929).

### Quality assessment

The quality of each study in terms of population and sampling methods, description of exposure and outcomes, and statistical adjustment of data, is summarized in Additional file 1: Appendix 1. All studies received a score of 6 or higher on the Newcastle-Ottawa Quality assessment scale and were considered to be of high methodological quality [12, 13, 19, 25, 26, 28, 30–38].

### Sensitivity analysis

To further identify the relationship between dietary patterns and CKD risk, we performed a sensitivity analysis. It showed that differences in age, sample size, race and study design had an important effect on the association between dietary patterns and CKD risk. When the highest category was compared with the lowest category of western-type dietary pattern, the western-type dietary pattern/CKD association was stronger in a small sample size, and subjects were more than 50 years old, and yellow and other. In addition, the inverse association was obvious for those in the highest compared with the lowest category of heavy drinking pattern in studies having a large sample size. Owing to these factors having a strong effect on the association between dietary patterns and CKD risk, their differences may partially explain the heterogeneity between studies (Table 3).

**Table 3:** Dietary patterns and CKD: sensitivity analysis

Study characteristic	Category	Healthy dietary pattern (95% CI)	Western-type dietary pattern (95% CI)	Heavy drinking pattern (95% CI)	Light-moderate drinking pattern (95% CI)
Age	> 50	0.78 (0.64, 0.97)	2.25 (1.42, 3.57)	0.66 (0.49, 0.88)	0.75 (0.69, 0.81)
	< 50	0.58 (0.49,0.70)	1.28 (0.42, 3.94)	0.67 (0.54, 0.84)	0.79 (0.70, 0.88)
Sample size	Large (> 5000)	0.71 (0.55, 0.91)	1.20 (0.70, 2.05)	0.67 (0.55, 0.81)	0.76 (0.71, 0.81)
	Small (< 5000)	0.67 (0.50, 0.88)	2.83 (1.39, 5.76)	0.65 (0.40, 1.05)	0.69 (0.49, 0.98)
Race	White	0.69 (0.51, 0.92)	1.79 (0.56, 5.74)	0.66 (0.49, 0.88)	0.75 (0.69, 0.81)
	Yellow and Other	0.69 (0.54, 0.88)	1.87 (1.28, 2.72)	0.67 (0.54, 0.84)	0.79 (0.70, 0.88)
Study design	Cross-sectional	0.70 (0.53, 0.91)	2.04 (1.15, 3.64)	______	________
	Cohort	0.69 (0.54, 0.88)	1.38 (1.18, 1.63)	0.67 (0.56, 0.80)	0.76 (0.71, 0.81)

## Discussion

Limited literature has investigated the link between dietary patterns and the risk of CKD in Chinese population [13, 19], and has yielded inconclusive results. To the best of our knowledge, the present study is the latest systematic review and meta-analysis on the association of dietary patterns with the risk of CKD. In the current study, the results showed that a healthy dietary pattern and alcohol drinking were associated with lower risk of CKD, whereas a Western-type dietary pattern was associated with higher risk of CKD. Data from 17 studies involving 149,958 participants were included in the present analysis.

In our analyses, a healthy dietary pattern was associated with lower risk of CKD. Our findings are in agreement with previous a study by Bach et al., reporting that a healthy dietary pattern was associated with a lower incidence of CKD [39]. however, almost all of included studies from previous meta-analysis were conducted in the United States or Iran. Thus,the results may not be extrapolated to other populations around the world. Besides, the previous meta-analysis was based only on cohort studies without other types of studies, e.g. randomized trails, and the results may have selection bias. Anywhere, there are several plausible explanations for the protective effect of healthy pattern on CKD. First, the healthy pattern tends to have high loadings of whole grains, fruit and vegetables, containing large amounts of dietary fiber. Some previous studies reported that high intake of dietary fiber was significantly associated with a decreased risk of CKD [40]. Although the exact mechanism remains unclear, dietary fiber may reduce the levels of inflammatory markers, e.g.interleukin 6(IL6), total homocysteine and C-reactive protein (CRP), which are common precursors to CKD [41]. Second, the protective effects of fruits and vegetables consumption against CKD may be related to their high concentration of folate. In a cross-sectional study conducted in Australia, folate intake (base on estimated average recommended) was associated with a 40–45% decreased risk of CKD [42]. Finally, vegetables and fruits are rich in antioxidants, for example vitamin C, vitamin E, and other carotenoids compounds. It has been widely agreed that antioxidants play an important role in reducing the risk of CKD [43].

The Western-type dietary pattern was significantly associated with higher risk of CKD. Our results were consistent with previous findings [19], showing that western pattern was related to an elevated risk of CKD. Xu et al. [19]studied the Western dietary pattern defined byred meats, poultry and organs, processed and cooked meat, eggs, seafood, cheese, fast foods, snacks, chocolates, alcoholic beverages, coffee that was positively associated with the risk of CKD. Due to the cross-sectional design of this study, Xu et al. cannot infer causality. Meanwhile, the participants were predominately recruited Hangzhou city, China and not a random sample of the general population. The findings cannot be generalizable to other populations. As we all know, the Western pattern is usually composed of red and/or processed meat, refined grains, sweets, high-fat dairy products, butter, potatoes and high-fat gravy. Thus, several plausible explanations have been proposed to elucidate the detrimental association of Western-type pattern with CKD risk. First, high intake of meat containing large amounts of saturated fat and cholesterol, was strongly associated with the development of CKD [43]. Second, processed meat and fast foods often contain high content of salt. Previous a study reported that high salt consumption is positively associated with the development of CKD [5]. Third, some previous studies have shown that western dietary pattern was associated with higher risk of obesity, hypertension and T2DM, all of these factors have been documented to be important risk factors for CKD [40]. Finally, soft drinks contain large amounts of fructose. Previous studies have shown that sugar consumption, especially in the form of fructose, was associated with an increased risk of kidney disease [44].

Light-moderate and heavy drinking patterns were associated with lower risk of CKD in the present meta-analysis. A previous systematic review and meta-analysis of alcohol consumption and the risk of renal damage demonstrated that high alcohol consumption was associated with a 17% decreased risk of CKD [45]. Besides, a 10-year longitudinal Japanese study including 123,764 adults with eGFR≥ 60 ml/min/1.73m^2^ showed that moderate alcohol consumption(< 20 g/d) was associated with a decreased risk of CKD [46]. Conversely, a prior study found no significant relationship between heavy drinking pattern and CKD in a middle-aged Japanese men [36]. Thus, the relationship between alcohol consumption and the risk of CKD remains controversial. In fact, alcohol consumption has been recognized as one of the major risk factors for poor health outcomes. In the current study, we observed an inverse association between light-moderate drinking pattern and the risk of CKD. There are several plausible explanations for this inverse association. First, an earlier study has demonstrated that moderate alcohol consumption can increase high-density lipoprotein (HDL) and plasma concentration of endogenous tissue-type plasminogen activator [47], thereby protecting against atherosclerosis, a major risk factor for CKD [46]. Second, polyphenols rich in red wine were widely believed to have beneficial health effects due to their anti-oxidant properties [39]. Studies have shown that long-term exposure to polyphenols may reduce kidney injury by induction of superoxidase dismutase, glutathione peroxidase, and catalase [48]. Third, moderate alcohol consumption has been found to be associated with decreased risk of coronary heart disease, which shares similar risk factor and pathophysiology with CKD [34, 49]. Meanwhile, we also observed an inverse relationship between heavy drinking pattern and the risk of CKD in this analysis. However, high alcohol consumption of more than two drinks per day was shown to increase the risk of ESRD in the American population [50]. As far as we know, excessive alcohol consumption could interfere with electrolyte and acid-base balance and body fluids, which can negatively affect kidney function [51]. Besides, previous studies have also shown that heavy alcohol consumption is positively associated with hypertension, one of the major risk factors for CKD [52]. Furthermore, excessive alcohol consumption have become a worldwide problem, with approximately 140 million people having alcoholism [53]. In short, heavy or excessive drinking should be discouraged.

In this meta-analysis, there was significant heterogeneity across the part of included studies, which may be attributed to differences in the ways that healthy and western-type dietary patterns were defined or dissimilar characteristics of individuals sticking to healthy/unhealthy in the East and West countries. Although we have tried to match the similar factor loadings as much as possible between included studies, the actual factor loading of the same foods within the similar dietary pattern was never exactly the same between studies [54]. There may also be other variables that cannot be explained, e.g. cooking methods. These variables may be culturally related and vary by race. In the sensitivity analysis, we also observed that differences in race had an important effect on the association between dietary patterns and CKD risk. Nevertheless, because of the relatively small number of included studies, we cannot to explore all potential sources of heterogeneity. Future studies could shed light on whether other variables, such as cooking methods, may influence the association between dietary patterns and CKD risk.

### Strengths and limitations

This systematic review and meta-analysis had several strengths. First, CKD diagnosis was confirmed by glomerular filtration rate (eGFR,in ml/min/1.73m^2^), and those with eGFR< 60 ml/min/1.73m^2^ or the presence of albuminuria were classified as having CKD, avoiding misdiagnosis bias. Second, this is the latest systematic review and meta-analysis on the association of dietary patterns with the risk of CKD. We not only have an update on the previous meta-analysis (Bach et al., 2019, ^39]^,but also further explore the association between drinking pattern and the risk of CKD. Besides, our findings also provide a clear evidence for the association between dietary patterns and CKD risk. Third, no signs of publication bias were evident in the funnel plot, and the statistical test for publication bias was non-significant. Nonetheless, the present meta-analysis also had some limitations that should be acknowledged. First, the principal limitation was the use of potentially biased evidence. There were some inconsistencies in the confounding factors adjusted among the included studies. As a consequence, the data included in our analyses may suffer from differing degrees of completeness and accuracy. Second, 7 of 17 studies were cross-sectional in term of study design, which are more prone to recall and selection bias, especially dietary recall bias, than a cohort design. Finally, the geographical regions covered in the present meta-analysis included Asia (China, Iran and Japan), Europe (England, Sweden and Netherlands), and United States. Thus, the results of this meta-analysis could not be expanded to all populations.

### Conclusion

In conclusion, this systematic review and meta-analysis indicated that a healthy dietary pattern and alcohol drinking were associated with lower risk of CKD, whereas a Western-type dietary pattern was associated with higher risk of CKD. These findings provide evidence of the role of dietary pattern in the development of CKD, and highlight the importance of higher vegetable, fruit and whole grains and lower red meat and high-fat diet in order to be associated with lower risk of CKD. Therefore, it makes sense to provide a scientific rationable for formulating dietary guidelines.

## Supplementary Information

**Additional file 1.**

## Data Availability

All the data in this meta-analysis are from published studies and we take responsibilities for the data integration process and the accuracy of the statistical analyses process.

## Abbreviations

CKD: Chronic kidney disease
OR: Odds ratio
CI: Confidence interval
BMI: Body mass index
WC: Waist circumference
WHR: Waist-hip ratio
SBP: Systolic blood pressure
DBP: Diastolic blood pressure
HR: Hazard ratios
RR: Relative risks
FFQ: Food frequency questionnaire
HDL: High- density lipoprotein
