# Supplementary material for: Dietary patterns and chronic kidney disease risk: a systematic review and updated meta-analysis of observational studies
Source: Nutr J. 2021 Jan 8;20:4. doi: 10.1186/s12937-020-00661-6 (PMC7796538; doi:10.1186/s12937-020-00661-6)
Supplement: Supplementary file 1 — Additional file 1. [file 12937_2020_661_MOESM1_ESM.doc]

**Appendix1.** Dietary patterns and CKD: Assessment of Study Quality

| **Studies** | **Selection** | | | |  | **Comparability** | |  | **Outcome** | | | **Score** |
| --- | --- | --- | --- | --- | --- | --- | --- | --- | --- | --- | --- | --- |
|  | 1 | 2 | 3 | 4 |  | 5A | 5B |  | 6 | 7 | 8 |  |
| Hu et al 2019 | * | * | * | * |  | * | * |  | * | * | * | ********* |
| Kurniawan et al 2019 | * |  | * | * |  | * |  |  | * | * | * | ******* |
| Yuzbashian et al 2018 | * | * | * | * |  | * | * |  | * | * | * | ********* |
| Xu et al 2020 | * |  |  | * |  | * |  |  | * | * | * | ****** |
| Shi et al 2018 | * | * |  | * |  | * | * |  | * | * | * | ******** |
| Asghari et al 2018 | * | * | * | * |  | * | * |  | * | * | * | ********* |
| Paterson et al 2018 | * |  |  | * |  | * |  |  | * |  | * | ***** |
| Lara et al 2019 | * | * | * | * |  | * |  |  | * | * | * | ******** |
| Rouhani Hossein et al 2019 | * |  |  | * |  | * |  |  | * | * | * | ****** |
| Mazidi et al 2018 | * |  | * | * |  | * |  |  | * | * | * | ******* |
| Huang et al 2013 | * | * | * | * |  | * | * |  | * | * | * | ********* |
| Eimery et al 2020 | * |  |  | * |  | * |  |  | * |  | * | ***** |
| Hu et al 2020 | * | * | * | * |  | * |  |  | * | * | * | ******** |
| Koning et al 2015 | * | * | * | * |  | * |  |  | * | * | * | ******** |
| Sato et al 2014 | * | * | * | * |  | * |  |  | * | * | * | ******** |
| Okada et al 2019 | * | * | * | * |  | * |  |  | * | * | * | ******** |
| Foster et al 2015 |  | * | * | * |  | * |  |  | * | * |  | ****** |
